# Improving hypertension control in patients at cardiovascular risk: the case for telmisartan-based therapy

**Published:** 2011

**Authors:** Aalbers J

**Affiliations:** Special Assignments Editor

Strategies to improve hypertension control are clearly warranted in light of the frequently expressed view that only 50% of treated hypertensive patients are able to meet their target blood pressure levels.

While data from the USA has shown improved control over recent years (Table 1), a study in 2008 of South African general medical practice (private sector) showed that 61% of patients reached their target blood pressure levels. If the even-stricter target blood pressure levels of 130/80 mmHg were applied to those patients with co-morbidities who needed to reach and maintain these lower levels, only 40% of patients were successfully treated.^1^

**Table 1:** TRENDS IN HYPERTENSION PREVALENCE, AWARENESS, TREATMENT AND CONTROL IN THE USA^*^

	1988–1991	1991–1994	1999–2000	2007–2008
Hypertension prevalence (%)	25.0	25.0	28.7	29.0
Awareness (%)	69.2	67.8	68.9	80.7
Treatment (%)	52.4	52.0	58.4	72.5
Control				
Among those treated (%)	46.9	43.6	53.1	69.1
Among all with hypertension (%)	24.6	22.7	31.0	50.1

^*^Data derived from Jajjar and Kotchen, copyright 2003, American Medical Association and Egan et al., copyright 2010, American Medical Association.

In primary-care, public-sector facilities in South Africa, special efforts to improve hypertension management have shown that 68% of patients treated for their hypertension can achieve their targeted blood pressure levels.^2^ Without these intensive programmes, however, hypertension control in the South African public sector is likely to be much less effective and lower than in the wellserviced private sector.

Reducing the cardiovascular and renal consequences of hypertension is dependent on sustained, long-term blood pressure control, implying that patient compliance is also a key factor for success. The physician’s choice of effective therapy will take this aspect fully into account and he/she will adopt approaches that will sustain patient compliance.

Modern therapeutic agents that block the renin–angiotensin–aldosterone system (RAAS) and protect target organs without causing compliance-reducing symptoms should be the first choice in at-risk patients with hypertension.

## Achieving sustained blood pressure control

**Once-a-day dosage**

Patients typically prefer to take their medication in the morning as part of their everyday routine. Compliance is improved by once-daily medication and physicians are keen to ensure that the prescribed antihypertensive medication meets the criteria of full 24-hour control and provides cover for the early morning rise in blood pressure. This rise in blood pressure is due to both orthostatic changes and the circadian rhythm of the RAAS system. It is also linked to an increased risk of cardiovascular events during the early morning hours.^3^

Accurate assessment of blood pressure control is determined by self-measurement of blood pressure or by automated ambulatory 24-hour measuring devices. The MICARDIS Community Access Trial of Telmisartan in the primary-care setting (MICCAT-2)4 study showed that telmisartan alone or in combination with HCTZ produced significant reductions in blood pressure, which extended into both day and night time. Telmisartan reduced systolic and diastolic blood pressure (SBP/DBP) by 17.2/10.^1^ mmHg in the first four hours post-awakening in patients whose early morning blood pressure rose more than 30 mmHg prior to therapy.

**ARB efficacy versus ACE inhibitors**

ARBs are a good choice for hypertensive patients with the metabolic syndrome (associated obesity) and there are compelling indications for their use in postmyocardial infarction, left ventricular hypertrophy, chronic kidney disease, type 2 diabetes with microalbuminuria or albuminuria for ACE-intolerant patients and for the secondary prevention of stroke.^5^ The evidence for therapeutic equivalence of telmisartan versus ACE inhibitors resides in direct major comparison trials with ramipril and perindopril. In the PRISMA-1 study (Prospective Randomised Investigation of the Safety and efficacy of MICARDIS versus ramipril) also conducted in South Africa, 1 613 hypertensive patients were treated either with temisartan 40–80 mg or ramipril (uptitrated from 2.5–10 mg) in the morning, and resulting blood pressure was evaluated using ambulatory blood pressure monitoring.

Telmisartan provided more effective blood pressure lowering in this study and was particularly more efficient in the last six hours of the 24-hour dosing interval. Similar results were obtained by PRISM-2, which was conducted in the USA and Canada. A pooled analysis of both trials also showed a greater blood pressure lowering with telmisartan (–14.1/–9.6 vs –11.1/–7.2 mmHg).^6^ In a double-blind study of telmisartan 80 mg versus perindopril 4 mg, similar results in blood pressure lowering were obtained but telmisartan resulted in lower diastolic blood pressures over the last eight hours of therapy. Other studies versus lisinopril produced similar results.

Telmisartan is the only ARB that has demonstrated therapeutic equivalence to the ACE inhibitor ramipril in hypertensive patients at increased vascular risk. The patient population in this study (ONTARGET) is of particular interest as it is representative of the majority of hypertensive patients seen in everyday clinical practice.

The findings from this study showed that telmisartan 80 mg per day was as efficacious as the proven dosage of ramipril (10 mg/day) in reducing risk of cardiovascular death, myocardial infarction, stroke and hospitalisation for heart failure in a broad cross section of highrisk cardiovascular patients. It achieved these results with far fewer side effects, resulting in significantly fewer patients discontinuing therapy.

**Choosing telmisartan over other ARBs: the evidence**

Pharmacological evidence of telmisartan’s efficacy in terms of blocking the angiotensin II type 1 receptor is accumulating. A recent Japanese study of constructed models of ARB molecules has found that the delta lock structure of telmisartan offers a superior fit to the receptor, compared to the other ARBs.^7^ This fit may explain the highest lipophilicity, the greatest volume distribution and the strongest binding affinity of telmisartan to the type 1 receptor when compared to other ARBs. This receptor affinity is likely to contribute to the clinical evidence for telmisartan’s greater blood pressure lowering compared to other ARBs, particularly losartan and valsartan.^8^-^10^

**bChoosing the combination of telmisartan + HCTZ**

Blood pressure control in some patients is ineffective with just monotherapy, and combinations of antihypertensive agents offer an opportunity to intensify treatment without adding to the pill load.

In African patients where there may be evidence of less involvement of the RAAS system, the initial choice in the public sector is often a diuretic. A recent study in KwaZulu-Natal looked at prescribing habits in 54 public-sector hospitals in this region and compared this to supply data and to the SA Hypertension Guidelines.^11^

It found that the most commonly used agents were diuretics (42%) and ACE inhibitors (27%) and that these prescriptions correlated well with supply data for these frequently used medications. Calcium channel blockers and beta-blocker usage was 6% each.

Clearly, as ARBs become more available in the public sector, the opportunities offered by the combination of telmisartan and HCTZ should receive wider use. Publicity given to the lack of dialysis facilities for kidney failure in the public sector in South Africa should also add to the imperative to improve hypertension control.

The blood pressure of patients who are at particular risk of cardiovascular disease, such as those who are obese or have type 2 diabetes, are often difficult to control. The SMOOTH study (Study of Micardis on Obese/Overweight Type 2 diabetes patients with HypErtension) showed the superior effect of telmisartan 80 mg plus HCTZ 12.5 mg, compared to valsartan 160 mg plus HCTZ 12.5 mg over 24 hours and especially in the early morning period.^12^

The elderly patient also frequently falls into a difficult-to-treat category. A study in this patient group recruited 1 000 patients over the age of 60 years with isolated systolic hypertension and compared control between telmisartan plus HCTZ and amlodipine plus HCTZ. Referred to as the ACTOS study,^13^ this showed the generally more effective blood pressure-lowering action of telmisartan in the last six hours of the dosing interval, and more effective systolic blood pressure lowering throughout the 24-hour treatment period and in the morning and evening periods.

A recent evaluation of more than 48 000 patients taking either a diuretic of a fixed-combination antihypertensive with a diuretic showed greatly improved compliance with the fixed combination (32 vs 50%, respectively). The fixed combination of the ARB/HCTZ achieved improved persistence and adherence to therapy even over the ACEI/HTCZ.

The wider use of telmisartan in moderate hypertension as an early therapy can be substantiated at both a pharmacological and clinical level.
